# Standing in unperturbed balance improves spatial memory and facilitates allocentric representation

**DOI:** 10.3389/fnbeh.2026.1895560

**Published:** 2026-10-09

**Authors:** Achille Pasqualotto, Jun Aoki, Kenji Suzuki, Hideki Kadone

**Affiliations:** 1 Faculty of Human Sciences, University of Tsukuba, Tsukuba, Japan; 2 Japan Society for the Promotion of Science (JSPS), Tokyo, Japan; 3 Institute of Systems and Information Engineering, University of Tsukuba, Tsukuba, Japan; 4 Institute of Medicine, University of Tsukuba, Tsukuba, Japan

**Keywords:** allocentric, egocentric, spatial memory, spatial representation, standing mobility device

## Abstract

Wheelchair users face numerous challenges, which might include experiencing the world from a sitting modality, thus resulting in poorer spatial representation. Our study is an initial exploration of this hypothesis that, to begin with, involved healthy participants using a novel mobility device for sitting *and* standing locomotion. According to the situations, this device allows for transitioning from the sitting to the standing modality and vice versa. Additionally, we adopted a “classical” methodology to investigate spatial representation. Participants [*N* = 40] saw a scene of real objects (a book, a teapot, a football, etc.) arranged in a large room. Crucially, a half of them was using the device in the sitting modality, and the other half was in the standing modality. Afterwards, a spatial memory task investigated participants’ spatial representation. More importantly, this task assessed whether the spatial representation was more egocentric (viewer-to-objects) or allocentric (objects-to-objects). Results showed that the standing group outperformed the sitting group [*p* < 0.001] and possessed a markedly allocentric spatial representation [*p* < 0.001], thus indicating that the standing group was better at grasping the spatial relations among objects. This set of results suggests that the higher viewpoint afforded by the standing modality provided more spatial information to the brain areas involved in spatial processing, thus resulting in a more robust spatial representation. Aside its theoretical relevance, this finding might have practical implications for improving independence and life quality of wheelchair-users.

## 1 Introduction

The novel mobility device for standing locomotion presented by Eguchi et al. (2018) is a promising tool to encourage independent mobility in wheelchair users. The main feature of this equipment is that users can easily transit from a sitting to a standing modality (see Figure 1), thus allowing users to interact with other individuals in a “canonical” manner (i.e., sitting when others are sitting and standing when others are standing).

**Figure 1:**
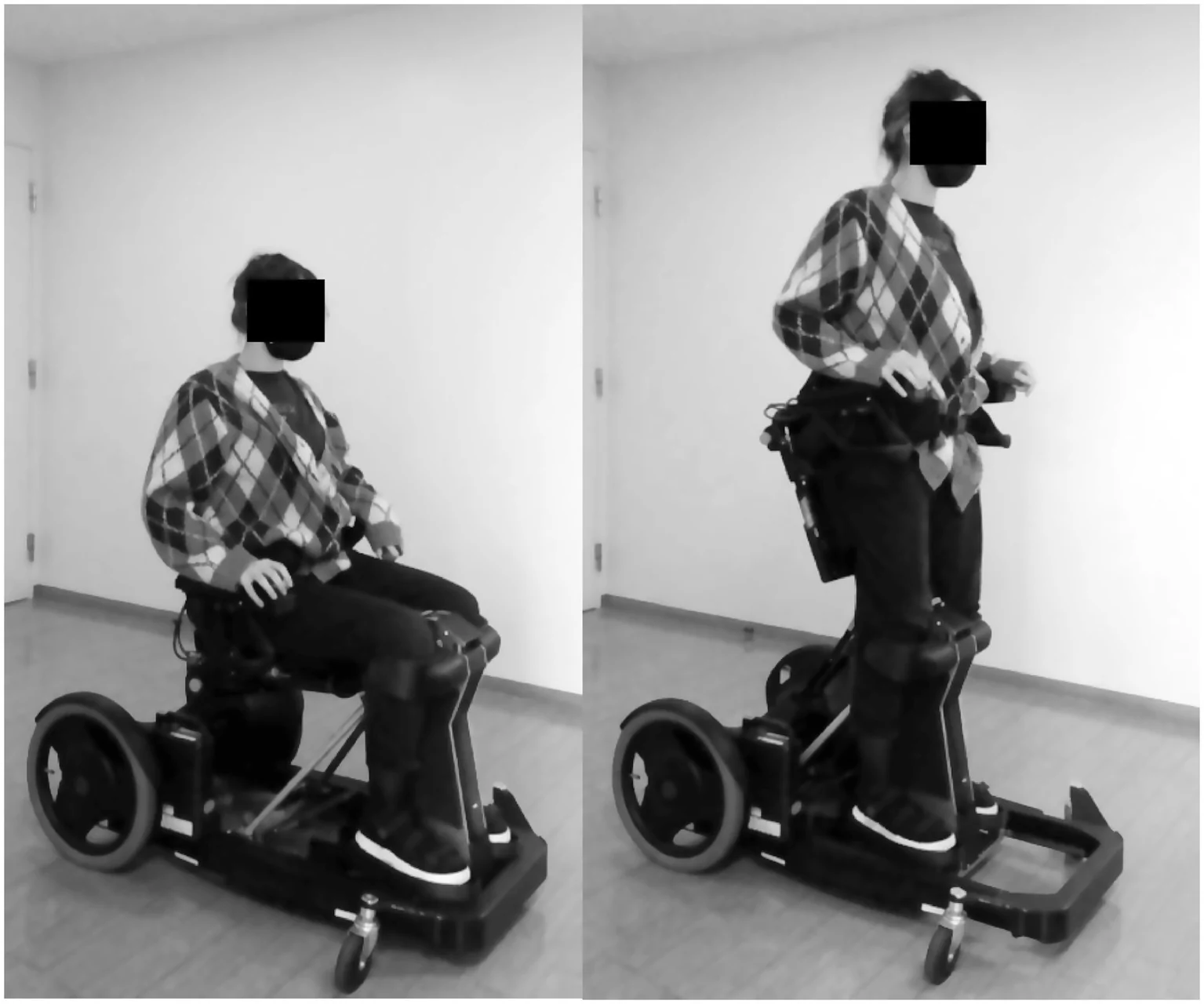
The mobility device used in this study in the sitting (left) and standing (right) modalities.

Aside from the intriguing possibility that the users of this device could improve their social interactions, our explorational study focused on the potential benefit on spatial representation (intended as spatial memory, Simons and Wang, 1998). We aim to test whether using the device in the standing modality, which provides a *higher* viewpoint, would benefit the spatial representation of the environment. This benefit may stem from the fact that a higher viewpoint facilitates the detection of the spatial relations *among* objects, thus resulting in a more robust allocentric spatial representation (Gori, et al., 2026; Klatzky, 1998; McNamara, 1986). For example, imagine that on a table there are several objects and you have to look at them to remember their positions. Your viewpoint could be either 5 cm above the table surface or 50 cm above. Your performance is likely to be more accurate the latter case, because the higher viewpoint provided more information about the spatial relations among objects.

However, the effect of sitting/standing on spatial representation (i.e., remembering the position of objects) can only be speculated because, at present, there are not formal investigations about this topic. For example, there is cognate research investigating the effect of horizontal vs. vertical spatial exploration on subsequent spatial memory. These studies generally reported that the advantage for remembering horizontal spaces is due to the fact that usually also spatial exploration follows horizontal routes (Hinterecker et al., 2018a, 2018b; Thibault et al., 2013), while rendering the vertical structure of a building easy to perceive (e.g., like in modern shopping malls) virtually eliminates the horizontal/vertical difference (Bock, 2025).

Studies on embodied cognition (Eerland et al., 2011; Ribeiro and Oliveira-Maia, 2025; Taya and Pasqualotto, 2025), provided evidence for a links between brain and body that effect our cognitive and perceptive processes. For example, there are studies focusing on the effect of cognitive tasks on balance (or cognition-on-balance, Maylor et al., 2001; Morioka et al., 2005; Nurwulan et al., 2015; Pellecchia, 2003) rather than on the effect of the sitting/standing modality on cognitive tasks (or balance-on-cognition, like ours). In general, cognition-on-balance studies reported that performing a cognitive task, for example writing a text message, disrupts balance (Nurwulan et al., 2015), although the magnitude of the disruption depends on the type of task (Maylor et al., 2001).

Indeed, studies like ours, focusing on the effect of the sitting/standing modality on cognitive tasks reported mixed results for non-spatial tasks, such as the Stroop task, which consist of telling the colour of the ink used to write words such as “red,” “green,” etc. (Caron et al., 2022; Smith et al., 2019; Šömen et al., 2023). Here, some studies reported the effect (e.g., Smith et al., 2019) and others did not (Caron et al., 2022). Likewise, in tasks with a spatial component, such as visual search (Labonté-LeMoyne et al., 2020; Zinchenko et al., 2025), attention deployment tasks (Abou Khalil et al., 2023), and spatial working memory tasks (Chen et al., 2018; Vander Velde et al., 2005) results are not conclusive in term of clarifying the effect of sitting/standing on cognitive performance. A recent study controlling for individual levels of fitness (Busch et al., 2025), reported that standing improved the performance in a perceptual task with spatial components (the Navon task, Navon, 1977).

The study by Busch et al. (2025) was quite revealing, because provided a possible explanation for the widespread mixed results reported by the studies investigating the effect of modality on spatial tasks. In fact, it suggested that results are affected by how good participants are at keeping their standing balance (for which the level of physical fitness is a proxy). Indeed, the study by Busch et al. (2025), which used the level of fitness of participants to statistically scale the results, is likely to have mitigated the effect of balance perturbations, because physical fitness reduces balance perturbations when standing.

Therefore, to study the effect of the sitting versus standing modality on the cognitive performance it is necessary to remove the confounding effect of balance perturbations during standing. Indeed, our mobility device eliminates this bias, because in the sitting and standing modalities the legs of participants are secured at the level of the knees, they wear a safety belt, and their arms lie on armrests (see Figure 1), thus effectively eliminating differences in balance stability between sitting and standing modalities.

With this ideal equipment, we embarked on a study investigating the effect of the sitting/standing modality on spatial memory inspired by the methods presented by the influential article by Mou and McNamara (2002) (see also Easton and Sholl, 1995) and successfully employed by us in previous works (Pasqualotto and Esenkaya, 2016; Pasqualotto et al., 2013). Briefly, these studies used a group of objects (scene) organised in rows and columns, and participants had to learn the position of each object. It was followed by a memory task called Judgement of Relative Direction (JRD, Rieser, 1989; Shelton and McNamara, 2001), where participants align themselves with an imaginary perspective and point to the required target object. For example: “Imagine that you are near the library’s main entrance and you are looking toward the fountain (this is the imaginary perspective), point to the Human Sciences’ building (this is the target).” A half of these imaginary perspectives were aligned with participants’ own view of the scene or parallel/perpendicular to it (egocentric), and a half with the parallel/perpendicular chessboard structure of the scene (allocentric). This is how we conceptualised egocentric and allocentric representations in the present work. However, others may consider as “egocentric” only participants’ own views of the scene, thus disregarding the other perspectives related to it (in Euclidean manner). Likewise, others may consider as “allocentric” only the “upright” view of the scene, thus disregarding the other perspectives related to it (in Euclidean manner). Therefore, our analyses and discussion will consider both ways to conceptualise egocentric and allocentric representations.

Therefore, our empirical questions are: when people stand on our device with an unperturbed balance, would they remember objects better than when sitting? How would they represent objects (egocentrically or allocentrically)? Based on the current literature on spatial tasks (e.g., Busch et al., 2025), we expect to find a benefit for the standing modality in terms of overall performance and allocentric processing (see the example of the objects on the table).

Finally, as technology and engineering advance and new devices like ours are created, the ability of these devices to match human needs must be assessed. Therefore, our study will address the open question of whether this mobility device can improve spatial representation, and in case of positive evidence, then investigations could be extended to wheelchair users.

## 2 Methods

### 2.1 Participants

Forty participants (20 males and 20 females, aged on average 25 years with standard deviation = 4.42) were recruited at the University of Tsukuba. Participants had normal or corrected-to-normal vision and were free from any conditions affecting their sitting and standing. The sample size was determined using G*Power 3.1.9.7 (Faul et al., 2009): “F-tests,” “ANOVA: Repeated measures, within-between interaction,” effect size = 0.24, and alpha = 0.05. Based on their arrival to the lab, participants were assigned to one of the two experimental groups in a random manner: Sitting (aged 22.95 ± 2.52 years, average height 163.55 ± 5.21 cm) and Standing (27.05 ± 6.32, 168.55 ± 10.63 cm). Independent-sample t-tests showed that the two groups did not significantly differ in terms of age [*t*(38) = 2.694, *p* = 0.010] or height [*t*(38) = 1.890, *p* = 0.66]. The study was approved by the Faculty of Systems and Information Engineering Research Ethics Committee (2022R655-3) and thus was compliant with the ethical guidelines of the Declaration of Helsinki. Participants received 1,000 Japanese yen vouchers for their participation.

### 2.2 Apparatus

The study was subdivided in learning and testing phases. The learning phase took place in a very large indoor space (30 x 20 m with ceilings about 10 m tall) without any furniture. There we set-up our apparatus consisting of seven real objects (see Figure 2A for their names) placed on white polystyrene “pedestals” 1.10 m tall. Objects were arranged as in Figure 2A (from now on “scene”). Additionally, we used the standing mobility device (Eguchi et al., 2018) oriented as in Figure 2 (“Viewing position”), on which participants sat/stood 1.5 m from the closest object. Aside the scene and the mobility device, the last piece of equipment was a movable curtain (4 m long and 2 m tall), which was used to occlude the scene.

**Figure 2:**
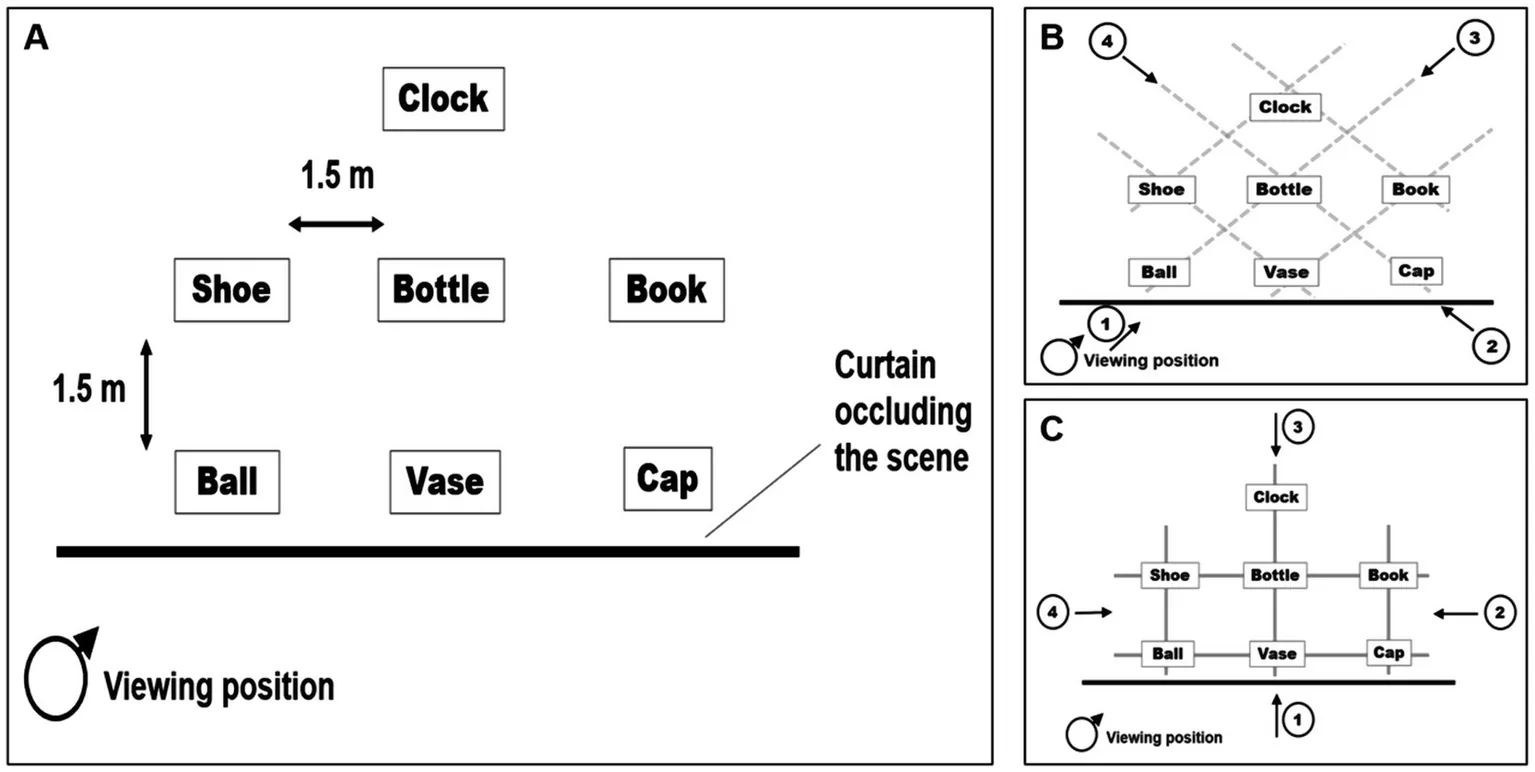
**(A)** The apparatus used in this study. Participants sat/stood at the viewing position oriented as indicated by the black arrow (‘nose’). **(B)** Depiction of the egocentric perspectives (numbers from-1-to-4), which are either the actual perspective experienced by participants (the ball-bottle line) or are related to it in an Euclidean manner (i.e., either parallel or perpendicular). There were six trials for each of the four egocentric perspectives (thus, 24 egocentric trials). For example, one of these trials was: “Imagine you are near the shoe and you are looking at the clock, point to the bottle” For the different conceptualisation of egocentric and allocentric representations, the “pure” egocentric perspective is perspective number 1. **(C)** Depiction of the allocentric perspectives (numbers from-1-to-4), which follow the Euclidean (parallel-perpendicular) structure of the scene. There were six trials for each of the four allocentric perspectives (thus, 24 allocentric trials). For example, one of these trials was: “Imagine you are near the vase and you are looking at the bottle, point to the book.” The “pure” allocentric perspective is perspective number 1. The resulting 48 trials were randomised for each participant, and we assured that when one of the eight perspectives was used in a trial then the following trial always used a different perspective.

The testing phase took place in an adjacent room using a joystick connected to a laptop computer running the MatLab (The MathWorks Inc., USA) programme used to collect pointing answers (in degrees) and reaction times. This task was a Judgment of Relative Direction (JRD, Mou and McNamara, 2002; Pasqualotto and Esenkaya, 2016; Pasqualotto et al., 2013), where participants had to take imaginary perspectives and pointed the joystick to the required targets. More specifically, they imagined themselves being in a particular position and looking toward a given direction, so that they could point to the required target. For example: “Imagine that you are near the library’s main gate (position) and you are looking toward the fountain (direction), point to the canteen (target).” Here, the seven objects of the scene were used in the JRD task. There were 48 trials; 24 were imaginary perspectives aligned with the egocentric views of the scene, with six trials for each of the four egocentric perspectives (see Figure 2B), and 24 were the imaginary perspectives aligned with the allocentric views of the scene, with six trials for each of the four allocentric perspectives (see Figure 2C). To construct the trials, for any given perspective first we chose the three objects near which participants were located (imagined positions). The neighbours of these “positioning” objects that were laying on the chosen perspective were the objects participants were looking at (imagined directions). For example, for Egocentric Perspective 1 (see Figure 2B), if *shoe*, *ball*, and *vase* were the objects indicating participants’ imagined positions, then their neighbours laying on Egocentric Perspective 1 were *clock*, *bottle*, and *book*, which created the position-direction pairs *shoe-clock*, *ball-bottle*, and *vase-book*. Finally, target objects were the remaining neighbours of the positioning objects. Each pair was used twice, thus creating a total of six unique trials for Egocentric Perspective 1 (the same procedure was used for the remaining perspectives). The performance in the JRD task is going to inform us on *how* participants represented the scene in their memory (egocentrically or allocentrically).

### 2.3 Procedure

After obtaining the informed consent and instructing the participants, we familiarised them with the JRD task. We used three examples involving landmarks around the university campus and the city centre. It was crucial that participants understood what the three objects used in each trial stood for (i.e., participants’ location, gaze direction, and the target object) and that the joystick had to be aimed performing a continuous moment without stopping halfway. To suit participants’ linguistic skills, the three examples were written in both English and Japanese, and a Japanese experimenter was always present during the experiment to answer questions or provide further explanations, if needed. Emphasis was put on response accuracy, while participants could “take their time” to respond, a policy maintained in the main experiment.

Then participants moved to the large space where the scene was arranged and sat/stood on the mobility device. In case they were sitting, we used cushions so that each participant’s eyes were at a height of around 150. In case they were standing, the height of the device was adjusted so that participants’ eyes were at a height of around 180 cm. During this time the scene was occluded by the curtain. Then participants wore a blindfold and experimenters folded the curtain. Finally, participants removed the blindfold and could see the scene for 30 s and tried to encode the position of the objects (Mou and McNamara, 2002; Pasqualotto and Esenkaya, 2016; Pasqualotto et al., 2013). The end of the 30 s, participants wore the blindfold and the experimenter occluded the scene with the curtain. To verify that participants had learnt the position of each object, they were requested to indicate the position of each object using their index finger. For example, “Where is the vase? Point it with your index finger,” “Where book?,” etc. This phase lasted until the position of all objects was correctly indicated for two consecutive runs. In most cases, participants needed just two runs to complete this verification (only two or three participants needed three runs). Additionally, this procedure familiarised participants with the objects’ names (see Figure 2) that were used in the testing phase of the experiment.

Afterwards, to minimise distractions during the JRD task, blindfolded participants were guided to the adjacent room by an experimenter. There blindfolded participants sat at the desk, the joystick was placed in front of them, and they performed the JRD task. In each trial, an experimenter slowly and with a neutral voice read aloud the trial, for example, “Imagine you are near the cap (3 s pause) and you are looking at the book (3 s pause), point to the bottle.” A “ping” sound indicted that participants could aim the joystick and it started the response timing as well. Timing terminated when the joystick was aimed at any direction. Thus, reaction times reflect both cognitive processing and motor execution. Yet, as all participants were instructed to aim the joystick in the same manner, it is likely that the time spent for the motor execution was quite similar across participants. No feedback about answers’ accuracy or timing was provided. To suit participants’ linguistic skills, trials were available in both English and Japanese. Experimenters were not blinded to group allocation. The duration of the pauses was measured with a watch.

The JRD task concluded the experiment that lasted around 50 min.

## 3 Results

For each trial we calculated the pointing errors in degrees (difference between the correct angle and the angle produced by the participant) and the reaction times (RT), in seconds. Then we performed a conservative data cleaning, where data points three standard deviations above/below the average of each participant were removed. This resulted in the removal of 21 data points from the pointing errors (or ~1% of the total), and 38 data points from RT (or ~2%). Finally, for each participant we calculated the average Pointing Error and RT (dependent variables) for the Sitting and Standing modalities (first independent variable) and the eight perspectives (four egocentric plus four allocentric, second independent variable), see Supplementary Tables S1–S4.

### 3.1 Pointing errors

Since our empirical question was to investigate the effect of modality and perspective, the four egocentric perspectives and (separately) the four allocentric perspectives were averaged to suit the 2 x 2 design (Modality [Sitting vs. Standing] x Perspective [Egocentric vs. Allocentric]). The Shapiro-Wilk test of normality was significant for three-out-of-four datasets [for all these *W*(20) < 0.881, *p* < 0.018]. Since non-normal datasets were the majority, we proceeded to normalise our data calculating their log_10_ (see Pasqualotto et al., 2020 for this procedure) and performed a two-way ANOVA.

The variable Modality was significant [*F*(1, 38) = 21.250, *p* < 0.001, *η*_*p*_^2^ = 0.359], indicating that Sitting (50.35 ± 21.51, or 1.66 ± 0.19 normalised) produced larger pointing errors than Standing (26.61 ± 15.96, or 1.35 ± 0.23), see Figure 3A.

**Figure 3:**
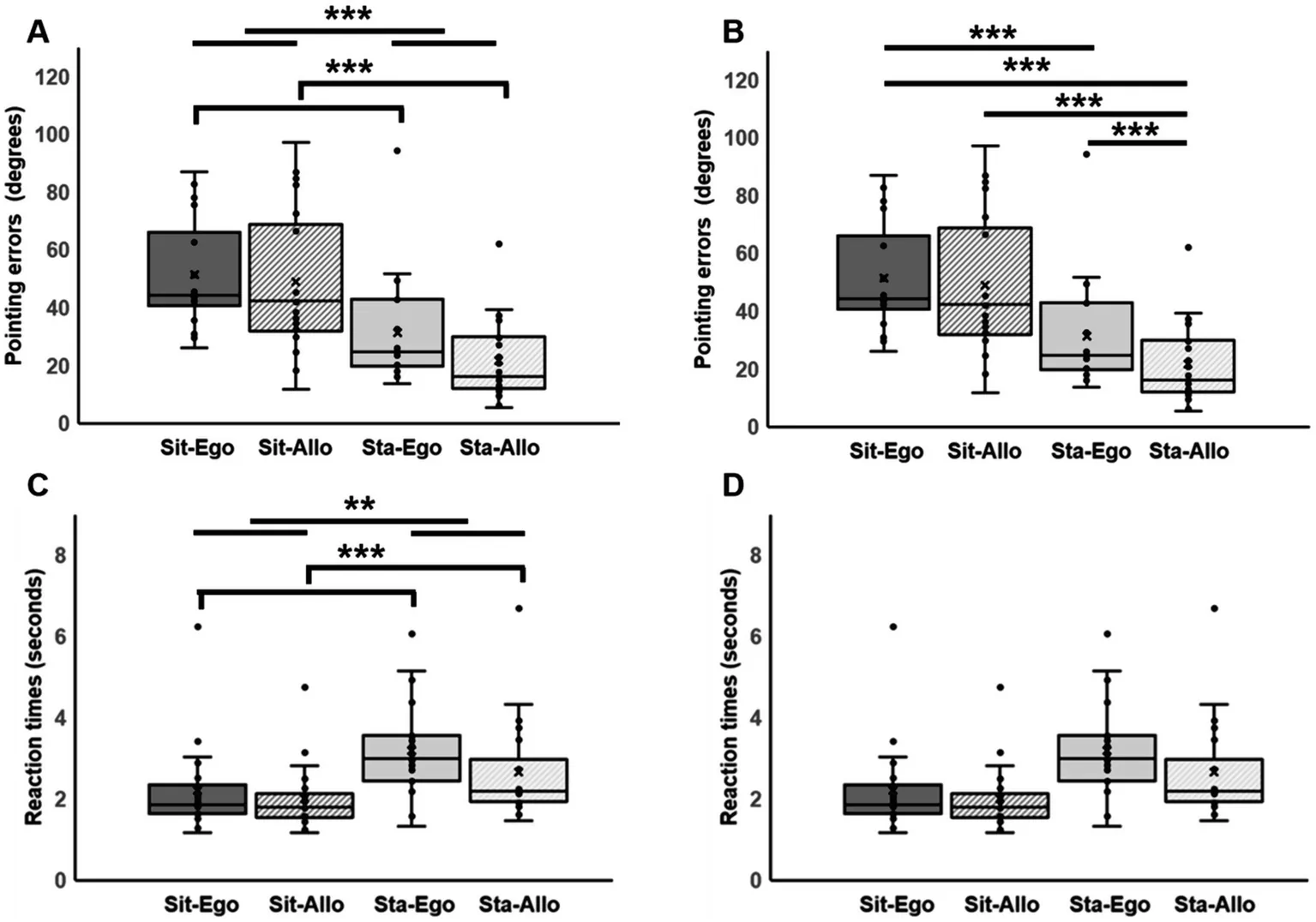
Pointing errors (above panels) and reaction times (below) across the four conditions; “Sit-Ego” is the Sitting group in the Egocentric condition, “Sit-Allo” is the Sitting group in the Allocentric condition, “Sta-Ego” is the Standing group in the Egocentric condition, and “Sta-Allo” is the Standing group in the Allocentric condition. The size of the bars indicates the quartile, the horizontal lines inside each bar indicate the median, and the “x” indicates the average. The whiskers indicate the minimum and maximum values (excluded outliers). The circles above the whiskers show the outliers. ** = “*p* < 0.010,” and *** = “*p* < 0.001.” For sake of clarity, main effects **(A,C)** and interactions **(B,D)** are depicted in separate panels. **(A)** Pointing errors with indicated the main effects of Modality and Perspective. **(B)** Pointing errors with indicated the interactions between Modality and Perspective. **(C)** Reaction times with indicated the main effects of Modality and Perspective. **(D)** Reaction times with indicated the interactions between Modality and Perspective.

The variable Perspective was also significant [*F*(1, 38) = 20.719, *p* < 0.001, *η*_*p*_^2^ = 0.353], where Egocentric perspectives triggered more errors (41.48 ± 12.83, or 1.60 ± 0.13) than Allocentric ones (35.48 ± 15.17, 1.51 ± 0.20), see Figure 3A.

The interaction between Modality and Perspective was significant [*F*(1, 38) = 6.260, *p* = 0.017, *η*_*p*_^2^ = 0.141] and was investigated using Bonferroni-corrected post-hoc analyses. Crucially, results indicated that for the Sitting group there was not a significant difference between Egocentric [51.57 ± 19.45, or 1.68 ± 0.16] and Allocentric [49.14 ± 25.30, or 1.63 ± 0.24] conditions [*p* = 0.932], while for the Standing group there was a significant difference between Egocentric [31.40 ± 18.88, or 1.44 ± 0.21] and Allocentric [21.83 ± 14.07, or 1.26 ± 0.28] conditions [*p* < 0.001]. These results may indicate a qualitative difference between the sitting and standing modalities, which will be discussed later. Additionally, the post-hoc comparisons indicated that the Sitting group in the Egocentric condition produced more errors than both the Egocentric and Allocentric conditions in the Standing group [*p* = 0.001 and *p* < 0.001, respectively]. Finally, the Sitting group in the Allocentric condition produced more errors than the Allocentric condition in the Standing group [*p* < 0.001]. The remaining post-hoc comparison was not significant [*p* = 0.072]; for a better grasp of the interactions, please refer to Figure 3B. The results relative to the different conceptualisation of egocentric and allocentric representations are in the Supplementary materials.

### 3.2 Reaction times

As for the pointing errors, the four egocentric perspectives and the four allocentric perspectives were averaged to fit our 2 x 2 design. The Shapiro-Wilk test of normality was significant for three-out-of-four datasets [for all these *W*(20) < 0.797, *p* < 0.001]. Non-normal datasets were the majority, and to perform the two-way ANOVA we normalised our data calculating their log_10_.

The variable Modality was significant [*F*(1, 38) = 8.107, *p* = 0.007, *η*_*p*_^2^ = 0.176], where Sitting (2.12 ± 0.95 s, or 0.30 ± 0.15 normalised) produced faster responses than Standing (2.93 ± 1.15, or 0.43 ± 0.16), see Figure 3C.

The variable Perspective was also significant [*F*(1, 38) = 16.257, *p* < 0.001, *η*_*p*_^2^ = 0.300], indicating that Egocentric perspectives required more time (4.08 ± 1.32, or 0.59 ± 0.14) than Allocentric ones (3.42 ± 1.24, or 0.51 ± 0.14), see Figure 3C.

The interaction between Modality and Perspective was not significant [*F*(1, 38) = 2.918, *p* = 0.096, *η*_*p*_^2^ = 0.071], see Figure 3D.

## 4 Discussion

The results of our explorational study indicated that the participants of the Standing group were more accurate at remembering the position of the objects (spatial representation) than the Sitting group. Additionally, allocentric perspectives produced a more accurate spatial memory performance than egocentric ones; is it because one group (Sitting or Standing) was particularly accurate with allocentric perspectives rather than egocentric ones? Indeed, the Standing group was particularly accurate with allocentric perspectives, which did not occur for the Sitting group (results relative to the interactions). This set of results strongly suggests that the standing modality improves spatial representation by triggering an allocentric spatial representation (i.e., remembering objects allocentrically). This result is in line with the portion of studies reporting that standing improves the performance in spatial tasks (Busch et al., 2025; Zinchenko et al., 2025), with the merit that our study provides results unaffected by balance perturbations, which we kept under control. As mentioned in the Introduction, a possible explanation of our results is that the higher observation point afforded by the standing modality provided more information about the objects and their spatial relation, thus resulting in a more accurate and allocentric spatial representation (perceptual mechanism). An alternative hypothesis in that standing per se increases the attention deployed toward external stimuli (Abou Khalil et al., 2024; Sawai et al., 2024), which has mild effects on job productivity as well (Chambers et al., 2019; Finch et al., 2017). In our case, paying more attention to the objects would result in a more accurate spatial representation as well (attentional mechanism).

Nevertheless, if the attentional mechanism were a suitable explanation of the results, this would have required the Standing group to *simply* outperform the Sitting group, with the variable Perspective (Egocentric vs. Allocentric) playing a non-significant role, which was not the case. In other words, if Standing increases attention then performance should similarly improve in egocentric and allocentric trials (compared with sitting). Instead, the Perspective (Egocentric vs. Allocentric) significantly effected participants’ performance and interacted with the Modality as well. Thus, a better explanation is that standing provided more information about the objects, thus resulting in an allocentric spatial representation (perceptual mechanism). Likewise, an explanation based on the Standing modality triggering novelty and arousal, would predict a general benefit for Standing with no effect of Perspective, which was not the case. Additionally, this hypothesis has the inherent problem of assuming that participants would be significantly more aroused by standing on our mobility device than by sitting on it. As a matter of fact, no sitting participant needed a wheelchair, and merely sitting on one is likely to have aroused them.

Additionally, our results relative to the reaction times suggest that the perceptual mechanism^1^ might be a better explanation of the accuracy results (instead of the “attentional” and “arousal” explanation). Indeed, the Standing group was slower to answer than the Sitting group, suggesting that more time was necessary to process the greater amount of information collected when Standing. This, mimics what we observed in a (single) object recognition task, where providing more visual information (stereoscopic information) resulted in slower object recognition (Pasqualotto and Hayward, 2009). Additionally, if Standing increased the attention toward the objects and/or the general arousal, we should have found faster responses when participants were standing, instead we found slower responses.

Overall, accuracy and reaction times seem affected by a speed-accuracy trade-off, where sitting participants are less accurate and faster, while standing participants are more accurate and slower. Yet, speed-accuracy trade-offs apply to the contexts where both speed and accuracy matter (Heitz, 2014; Karşılar, et al., 2014), which was not our case. As our primary interest was spatial representation, our participants were not required to be “as fast and as accurate as possible,” but just “accurate.” Thus, they could take the time they needed to solve each trial. Although this phenomenon requires further investigation, it is unlikely that speed-accuracy trade-off is involved in our study.

Although our data support the perceptual mechanism, discriminating for alternative mechanisms deserves a formal investigation. An ideal follow-up study should replicate the current study cancelling the differences in viewpoint height but maintaining modality differences (sitting/standing). Indeed, the settings of the mobility device could be adjusted so that sitting and standing participants will experience the same viewpoint height. Based, on what we wrote above, we expect that this procedure would eliminate the performance difference between sitting and standing.

In sum, accuracy results showed that the Standing group was more accurate with allocentric perspectives, while this did not happen for the Sitting group. This strongly suggests that the standing modality triggered allocentric spatial representation.

However, considering the different conceptualisation of egocentric and allocentric representations, the Standing modality appears to improve not just the allocentric perspectives (“pure” and “other”) but, surprisingly, also the “pure” egocentric perspective as well (Figure 2B, Perspective number 1, see also Supplementary materials). Although this conceptualisation is somewhat “a posteriori,” it highlights the benefit for the standing modality compared to the sitting.

Although a confirmatory study testing wheelchair users would be necessary, based on the current results, it is likely that our device will improve spatial knowledge also in this population, with the potential of improving their self-confidence when dealing with spatial matters, and in turn their independence and wellbeing. Another expansion of this explorational study would be testing our device in a study involving movement. Indeed, so far, we considered static spatial learning only, but usually we navigate across the environment and when we do so we *update* the position of objects to consider our own movement. Therefore, an ideal follow-up study would test spatial updating. More precisely, using the same setup, and using the mobility device in either the sitting or standing modalities, participants will view the objects and then *move* to another position to perform the spatial memory task from that new viewpoint. Results will tell us which modality is more effective for spatial updating and how objects will be represented.

In conclusion, to answer to the questions opened in the Introduction, we found that when people stand on our device and their balance is supported, then they remember objects better than when they are sitting, and they represent objects more allocentrically.

## Data Availability

The datasets presented in this study can be found in online repositories. The names of the repository/repositories and accession number(s) can be found at: https://osf.io/mceu8/overview.

## Footnotes

1 Higher viewpoint, hence more information was collected, hence an allocentric spatial representation was formed, hence a better performance in allocentric trials was recorded.

## References

[ref1] Abou Khalil G. Doré-Mazars K. Legrand A. (2023). Is better selective attention while standing possible without cost on postural sway?. Q. J. Exp. Psychol. 76, 1852–1861. doi: 10.1177/17470218221126591, 36073991

[ref2] Abou Khalil G. Doré-Mazars K. Legrand A. (2024). Stand up to better pay attention, sit down to better subtract: a new perspective on the advantage of cognitive-motor interactions. Psychol. Res. 88, 735–752. doi: 10.1007/s00426-023-01890-0, 37904007

[ref3] Bock O. (2025). Cognitive representations of multilevel buildings: two- or three-dimensional?. Exp. Brain Res. 243:article ID 202. doi: 10.1007/s00221-025-07136-2 40887528

[ref4] Busch N. Zinchenko A. Halle M. Geyer T. (2025). Withstand control: standing posture differentially affects space-based and feature-based cognitive control through enhanced physiological arousal. Sci. Rep. 15:26347. doi: 10.1038/s41598-025-11692-6, 40685471 PMC12277459

[ref5] Caron E. E. Marusich L. R. Bakdash J. Z. Ballotti R. J. Tague A. M. Carriere J. S. . (2022). The influence of posture on attention. Exp. Psychol. 69, 295–307. doi: 10.1027/1618-3169/a000567, 36809160 PMC10102972

[ref6] Chambers A. J. Robertson M. M. Baker N. A. (2019). The effect of sit-stand desks on office worker behavioral and health outcomes: a scoping review. Appl. Ergon. 78, 37–53. doi: 10.1016/j.apergo.2019.01.015, 31046958

[ref7] Chen Y. Yu Y. Niu R. Liu Y. (2018). Selective effects of postural control on spatial vs. nonspatial working memory: a functional near-infrared spectral imaging study. Front. Hum. Neurosci. 12:243. doi: 10.3389/fnhum.2018.00243, 29950981 PMC6008320

[ref8] Easton R. D. Sholl M. J. (1995). Object-array structure, frames of reference, and retrieval of spatial knowledge. J. Exp. Psychol. Learn. Mem. Cogn. 21, 483–500. doi: 10.1037//0278-7393.21.2.483 7738511

[ref9] Eerland A. Guadalupe T. Zwaan R. (2011). Leaning to the left makes the Eiffel Tower seem smaller: posture-modulated estimation. Psychol. Sci. 22, 1511–1514. doi: 10.1177/0956797611420731, 22123776

[ref10] Eguchi Y. Kadone H. Suzuki K. (2018). Standing mobility device with passive lower limb exoskeleton for upright locomotion. IEEE-ASME T. Mech. 23, 1608–1618. doi: 10.1109/TMECH.2018.2799865

[ref11] Faul F. Erdfelder E. Buchner A. Lang A. G. (2009). Statistical power analyses using G*Power 3.1: tests for correlation and regression analyses. Behav. Res. Methods 41, 1149–1160. doi: 10.3758/BRM.41.4.1149, 19897823

[ref12] Finch L. E. Tomiyama A. J. Ward A. (2017). Taking a stand: the effects of standing desks on task performance and engagement. Int. J. Environ. Res. Public Health 14:article ID 939. doi: 10.3390/ijerph14080939, 28825655 PMC5580641

[ref13] Gori M. Amadeo M. B. Bremner A. J. (2026). The development of spatial perception with and without visual experience. Nat. Rev. Psychol. 5, 80–92. doi: 10.1038/s44159-025-00516-z

[ref14] Heitz R. P. (2014). The speed-accuracy tradeoff: history, physiology, methodology, and behavior. Front. Neurosci. 8:150. doi: 10.3389/fnins.2014.00150, 24966810 PMC4052662

[ref15] Hinterecker T. Leroy C. Zhao M. Butz M. V. Bülthoff H. H. Meilinger T. (2018a). No advantage for remembering horizontal over vertical spatial locations learned from a single viewpoint. Mem. Cognit. 46, 158–171. doi: 10.3758/s13421-017-0753-9 PMC5758721 28875474

[ref16] Hinterecker T. Pretto P. de Winkel K. N. Karnath H. O. Bülthoff H. H. Meilinger T. (2018b). Body-relative horizontal-vertical anisotropy in human representations of traveled distances. Exp. Brain Res. 236, 2811–2827. doi: 10.1007/s00221-018-5337-9 PMC6153888 30030590

[ref17] Karşılar H. Simen P. Papadakis S. Balcı F. (2014). Speed accuracy trade-off under response deadlines. Front. Neurosci. 8:248. doi: 10.3389/fnins.2014.00248, 25177265 PMC4133757

[ref18] Klatzky R. L. (1998). “Allocentric and eegocentric spatial representations: definitions, distinctions, and interconnections,” in Spatial Cognition: An Interdisciplinary Approach to Representing and Processing Spatial Knowledge, eds. Freksa C. Habel C. Wender K. F.. Berlin: Springer, 1–17.

[ref19] Labonté-LeMoyne E. Jutras M. A. Léger P. M. Sénécal S. Fredette M. Begon M. . (2020). Does reducing sedentarity with standing desks hinder cognitive performance? Hum. Factors 62, 603–612. doi: 10.1177/0018720819879310, 31593493

[ref20] Maylor E. A. Allison S. Wing A. M. (2001). Effects of spatial and nonspatial cognitive activity on postural stability. Br. J. Psychol. 92, 319–338. doi: 10.1348/000712601162211, 11417784

[ref21] McNamara T. P. (1986). Mental representations of spatial relations. Cogn. Psychol. 18, 87–121. doi: 10.1016/0010-0285(86)90016-2 3948491

[ref22] Morioka S. Hiyamizu M. Yagi F. (2005). The effects of an attentional demand tasks on standing posture control. J. Physiol. Anthropol. Appl. Human Sci. 24, 215–219. doi: 10.2114/jpa.24.215 15930809

[ref23] Mou W. McNamara T. P. (2002). Intrinsic frames of reference in spatial memory. J. Exp. Psychol. Learn. Mem. Cogn. 28, 162–170. doi: 10.1037/0278-7393.28.1.162 11827078

[ref24] Navon D. (1977). Forest before trees: the precedence of global features in visual perception. Cogn. Psychol. 9, 353–383. doi: 10.1016/0010-0285(77)90012-3

[ref25] Nurwulan N. R. Jiang B. C. Iridiastadi H. (2015). Posture and texting: effect on balance in young adults. PLoS One 10:e0134230. doi: 10.1371/journal.pone.0134230, 26230323 PMC4521688

[ref26] Pasqualotto A. Esenkaya T. (2016). Sensory substitution: the spatial updating of auditory scenes “mimics” the spatial updating of visual scenes. Front. Behav. Neurosci. 10:79. doi: 10.3389/fnbeh.2016.00079, 27148000 PMC4838627

[ref27] Pasqualotto A. Hayward W. G. (2009). A stereo disadvantage for recognizing rotated familiar objects. Psychon. Bull. Rev. 16, 832–838. doi: 10.3758/PBR.16.5.832 19815785

[ref28] Pasqualotto A. Ng M. Tan Z. Y. Kitada R. (2020). Tactile perception of pleasantness in relation to perceived softness. Sci. Rep. 10, 1–10. doi: 10.1038/s41598-020-68034-x PMC7341757 32636415

[ref29] Pasqualotto A. Spiller M. J. Jansari A. S. Proulx M. J. (2013). Visual experience facilitates allocentric spatial representation. Behav. Brain Res. 236, 175–179. doi: 10.1016/j.bbr.2012.08.042, 22960256

[ref30] Pellecchia G. L. (2003). Postural sway increases with attentional demands of concurrent cognitive task. Gait Posture 18, 29–34. doi: 10.1016/s0966-6362(02)00138-8, 12855298

[ref31] Ribeiro C. Oliveira-Maia A. J. (2025). From cognition in the body to the body in cognition. Curr. Biol. 35, R967–R973. doi: 10.1016/j.cub.2025.08.062, 41118740

[ref32] Rieser J. J. (1989). Access to knowledge of spatial structure at novel points of observation. J. Exp. Psychol. Learn. Mem. Cogn. 15, 1157–1165. doi: 10.1037//0278-7393.15.6.1157, 2530309

[ref33] Sawai S. Murata S. Sakano Y. Fujikawa S. Yamamoto R. Shizuka Y. . (2024). Dominance of attentional focus: a comparative study on its impact on standing postural control in healthy younger and older adults. Front. Hum. Neurosci. 18:1384305. doi: 10.3389/fnhum.2024.1384305, 38988825 PMC11233467

[ref34] Shelton A. L. McNamara T. P. (2001). Visual memories from nonvisual experiences. Psychol. Sci. 12, 343–347. doi: 10.1111/1467-9280.00363 11476104

[ref35] Simons D. J. Wang R. F. (1998). Perceiving real-world viewpoint changes. Psychol. Sci. 9, 315–320. doi: 10.1111/1467-9280.00062

[ref36] Smith K. C. Davoli C. C. Knapp W. H. 3rd Abrams R. A. (2019). Standing enhances cognitive control and alters visual search. Atten. Percept. Psychophys. 81, 2320–2329. doi: 10.3758/s13414-019-01723-6, 31044397

[ref37] Šömen M. M. Peskar M. Wollesen B. Gramann K. Marusic U. (2023). Does standing up enhance performance on the Stroop task in healthy young adults? A systematic review and meta-analysis. Int. J. Environ. Res. Public Health 20:2319. doi: 10.3390/ijerph20032319, 36767687 PMC9915369

[ref38] Taya S. Pasqualotto A. (2025). The frog hand illusion: distortion of hand shape in inverted presentation. i-Perception 16, 1–9. doi: 10.1177/20416695251319270, 40026673 PMC11869244

[ref39] Thibault G. Pasqualotto A. Vidal M. Droulez J. Berthoz A. (2013). How does horizontal and vertical navigation influence spatial memory of multifloored environments? Atten. Percept. Psychophys. 75, 10–15. doi: 10.3758/s13414-012-0405-x, 23238988

[ref40] Vander Velde T. J. Woollacott M. H. Shumway-Cook A. (2005). Selective utilization of spatial working memory resources during stance posture. NeuroReport 16, 773–777. doi: 10.1097/00001756-200505120-00023, 15858423

[ref41] Zinchenko A. Busch N. Dodwell G. Geyer T. (2025). Withstand context: standing posture improves contextual cueing in challenging visual search. Psychophysiology 62:e70108. doi: 10.1111/psyp.70108, 40677214 PMC12272028

