# Supplementary material for: Standing in unperturbed balance improves spatial memory and facilitates allocentric representation
Source: Front Behav Neurosci. 2026 Oct 9;20:1895560. doi: 10.3389/fnbeh.2026.1895560 (PMC13690935; doi:10.3389/fnbeh.2026.1895560)
Supplement: Supplementary file 1 [file Data_Sheet_1.DOCX]

Standing in unperturbed balance improves spatial memory and facilitates allocentric representation

Achille Pasqualotto^1, 4^, Jun Aoki^2^, Kenji Suzuki^2^, and Hideki Kadone^3^

^1^Institute of Human Sciences, University of Tsukuba, Japan

^2^Institute of of Systems and Information Engineering, University of Tsukuba, Japan

^3^Institute of Medicine, University of Tsukuba, Japan

^4^Japan Society for the Promotion of Science (JSPS), Tokyo, Japan

Corresponding author:

Achille Pasqualotto, Faculty of Human Sciences, University of Tsukuba, 1-1-1 Tennodai, Tsukuba-shi, Ibaraki-ken, Japan

Table S1: Pointing errors in degrees relative to the *Sitting* group across the four egocentric (Ego.) and the four allocentric (Allo.) perspectives. The numbers after “Ego.” and “Allo.” are referring to the perspectives indicated in Figure 2B (for egocentric perspectives) and Figure 2C for allocentric perspectives. For example, here “Ego. 1” is perspective number 1 on Figure 2B, here “Allo. 1” is perspective number 1 on Figure 2C, and so on.

| Ego. 1 | Allo. 1 | Ego. 2 | Allo. 2 | Ego. 3 | Allo. 3 | Ego. 4 | Allo. 4 |
| --- | --- | --- | --- | --- | --- | --- | --- |
| 49.02 | 42.56 | 54.52 | 54.12 | 52.83 | 49.23 | 49.90 | 50.65 |
| (27.44) | (23.22) | (25.30) | (35.32) | (18.49) | (23.02) | (23.89) | (30.38) |

Table S2: Pointing errors in degrees relative to the *Standing* group across the four egocentric (Ego.) and the four allocentric (Allo.) perspectives. The numbers after “Ego.” and “Allo.” are referring to the perspectives indicated in Figure 2B (for egocentric perspectives) and Figure 2C for allocentric perspectives.

| Ego. 1 | Allo. 1 | Ego. 2 | Allo. 2 | Ego. 3 | Allo. 3 | Ego. 4 | Allo. 4 |
| --- | --- | --- | --- | --- | --- | --- | --- |
| 27.74 | 24.23 | 30.96 | 23.50 | 34.37 | 22.06 | 32.52 | 17.52 |
| (21.29) | (23.29) | (25.25) | (20.94) | (20.10) | (13.67) | (19.15) | (9.61) |

Table S3: Reaction times in s relative to the *Sitting* group across the four egocentric (Ego.) and the four allocentric (Allo.) perspectives. The numbers after “Ego.” and “Allo.” are referring to the perspectives indicated in Figure 2B (for egocentric perspectives) and Figure 2C for allocentric perspectives.

| Ego. 1 | Allo. 1 | Ego. 2 | Allo. 2 | Ego. 3 | Allo. 3 | Ego. 4 | Allo. 4 |
| --- | --- | --- | --- | --- | --- | --- | --- |
| 2.04 | 2.02 | 2.35 | 2.14 | 2.27 | 1.99 | 2.24 | 1.93 |
| (1.12) | (0.98) | (1.06) | (0.95) | (1.11) | (0.93) | (1.56) | (0.86) |

Table S4: Reaction times in s relative to the *Standing* group across the four egocentric (Ego.) and the four allocentric (Allo.) perspectives. The numbers after “Ego.” and “Allo.” are referring to the perspectives indicated in Figure 2B (for egocentric perspectives) and Figure 2C for allocentric perspectives.

| Ego. 1 | Allo. 1 | Ego. 2 | Allo. 2 | Ego. 3 | Allo. 3 | Ego. 4 | Allo. 4 |
| --- | --- | --- | --- | --- | --- | --- | --- |
| 2.69 | 2.33 | 3.13 | 2.81 | 3.65 | 2.87 | 3.28 | 2.68 |
| (1.30) | (1.26) | (1.21) | (1.45) | (1.70) | (1.49) | (1.02) | (1.18) |

Additional analyses and discussion

We analysed our data considering the different conceptualisation of egocentric/allocentric representations, where pointing errors from the “pure” egocentric perspective (Perspective 1 on Figure 2B), the pure allocentric (Perspective 1 on Figure 2C), “other” egocentric (Perspectives 2, 3, and 4 on Figure 2B), and other allocentric perspectives (Perspectives 2, 3, and 4 on Figure 2C) were separately analysed. This analysis closely resembles that by Mou and McNamara (2002, Experiment 2). As they did not have sitting and standing participants, we ran two separate one-way ANOVA, one for the Sitting group and one for the Standing group, on the variable Perspective (pure egocentric/pure allocentric/other egocentric/other allocentric).

For the Sitting group, the Shapiro-Wilk test of normality was significant for one-out-of-four datasets [*W*(20) = 0.878, *p* = 0.016]. Since normal datasets were the majority, we proceeded with the one-way ANOVA. The variable Perspective was not significant [*F*(2.677, 50.865) = 2.376, *p* < 0.087, *η_p_^2^* = 0.111], indicating that for the Sitting group the perspectives used in the memory test did significantly affect pointing errors (pure egocentric = 49.02±27.44 degrees, pure allocentric = 42.56±23.22, other egocentric = 52.42±18.80, and other allocentric 51.33±27.18).

For the Standing group, the Shapiro-Wilk test of normality was significant for three-out-of-four datasets [for all these *W*(20) < 0.775, *p* < 0.001]. Since non-normal datasets were the majority, we proceeded to normalise our data calculating their log_10_ and performed a one-way ANOVA. The variable Perspective was significant [*F*(2.260, 42.947) = 8.284, *p* < 0.001, *η_p_^2^* = 0.304], indicating that, unlike the Sitting group, the performance of the Standing group was significantly affected by the perspectives used in the memory trials. Bonferroni-corrected post-hoc analyses indicated significant differences between pure egocentric (27.74±21.29 degrees, or 1.35±0.27 normalised) and other egocentric (32.62±18.84, or 1.46±0.2) [*p* = 0.045], between pure allocentric (24.23±23.29, or 1.24±0.35) and other egocentric [*p* = 0.006], and between other allocentric (21.03±12.33, or 1.25±0.27) and other egocentric [*p* < 0.001]. The remaining three comparisons were not significant.

In sum, the results of these additional analyses suggested that, whereas the Sitting group is unaffected by the type of perspective used in the spatial memory trials, the Standing group is more accurate in the allocentric trials (both ‘pure’ and ‘other’), and in the pure egocentric trials. Therefore, according to this different conceptualisation of egocentric and allocentric repreparations, the benefit for the Standing modality seems to include the allocentric as well as the pure egocentric representations.
